# Diffusion-weighted imaging for assessment of synovial inflammation in juvenile idiopathic arthritis: a promising imaging biomarker as an alternative to gadolinium-based contrast agents

**DOI:** 10.1007/s00330-017-4876-y

**Published:** 2017-06-12

**Authors:** Anouk M. Barendregt, E. Charlotte van Gulik, Cristina Lavini, Charlotte M. Nusman, J. Merlijn van den Berg, Dieneke Schonenberg-Meinema, Koert M. Dolman, Taco W. Kuijpers, Robert Hemke, Mario Maas

**Affiliations:** 1 Department of Radiology, Academic Medical Center/University of Amsterdam, Meibergdreef 9, 1105 AZ Amsterdam, The Netherlands; 2 Department of Pediatric Hematology, Immunology, Rheumatology and Infectious Disease, Emma Children’s Hospital, Academic Medical Center/University of Amsterdam, Meibergdreef 9, 1105 AZ Amsterdam, The Netherlands; 3 Department of Pediatric Rheumatology, Reade, Dr. Jan van Breemenstraat 2, Amsterdam, The Netherlands; 4 Department of Pediatric Rheumatology, Onze Lieve Vrouwe Gasthuis West, Jan Tooropstraat 164, Amsterdam, The Netherlands; 5 Department of Pediatric Rheumatology, Onze Lieve Vrouwe Gasthuis Oost, Oosterpark 9, Amsterdam, The Netherlands

**Keywords:** Arthritis, Diffusion magnetic resonance imaging, Magnetic resonance imaging, Juvenile arthritis, Knee joint

## Abstract

**Objectives:**

To compare dynamic-contrast-enhanced MRI (DCE) and diffusion-weighted imaging (DWI) in quantifying synovial inflammation in juvenile idiopathic arthritis (JIA).

**Methods:**

Regions of interest (ROI) were drawn in the synovium of JIA patients on T1 DCE and T2 DWI, followed by extraction of the maximum enhancement (ME), maximum initial slope (MIS), time to peak (TTP), % of different time intensity curve shapes (TIC) and apparent diffusion coefficient (ADC) of the ROIs. Mann-Whitney-U test was used for comparing parameters between MRI-active and -inactive patients (defined by the juvenile arthritis MRI scoring system). Spearman’s rank was used to analyse the correlation between DCE and DWI.

**Results:**

Thirty-five JIA patients (18 MRI active and 17 MRI inactive) were included. Median age was 13.1 years and 71% were female. ME, MIS, TTP, % TIC 5 and ADC were significantly different in MRI-active versus MRI-inactive JIA with median ADC 1.49 × 10^-3^mm^2^/s in MRI-active and 1.25 × 10^-3^mm^2^/s in MRI-inactive JIA, *p* = 0.001, 95% confidence interval of difference in medians =0.11-0.53 × 10^-3^mm^2^/s. ADC correlated to ME, MIS and TIC 5 shapes (*r* = 0.62, *r* = 0.45, *r* = -0.51, respectively, all *p* < 0.05).

**Conclusions:**

Similar to DCE parameters, DWI-derived ADC is significantly different in MRI-active JIA as compared to MRI-inactive JIA. The non-invasiveness of DWI combined with its possibility to detect synovial inflammation shows the potential of DWI.

***Key Points*:**

• *MRI can quantify: dynamic contrast*-*enhanced and diffusion*-*weighted MRI can quantify synovitis*

• *Both DWI and DCE can differentiate active from inactive JIA*

• *The DWI*-*derived apparent diffusion coefficient* (*ADC*) *is higher in active JIA*

• *DWI is non*-*invasive and thus safer and more patient*-*friendly*

• *DWI is a potentially powerful and non*-*invasive imaging biomarker for JIA*

## Introduction

Interest in the role of magnetic resonance imaging (MRI) in juvenile idiopathic arthritis (JIA) is increasing, especially in detecting active disease and recognising early changes in JIA [1–3]. JIA is characterised by auto-immune-mediated synovial inflammation, which can expand to bone and cartilage and give rise to significant morbidity [4]. Therefore, it is important to initiate treatment in a timely manner and monitor treatment effects closely to prevent irreversible damage to the joints.

Early changes in JIA, e.g. synovial hypertrophy and effusion, are reliably visualised on contrast-enhanced MRI [3, 5, 6]. Currently, the semi-quantitative Juvenile Arthritis MRI Scoring system (JAMRIS) is the only validated MRI score for assessing disease activity of the knee in JIA [7]. The system uses morphological sequences; for example for scoring the thickness of the synovial membrane a contrast-enhanced T1 sequence is used. In contrast to traditional morphological sequences, MRI sequences such as dynamic contrast-enhanced MRI (DCE) and diffusion-weighted imaging (DWI) can derive measures that reflect inflammatory responses in the synovium [i.e. synovial hyperplasia and hypervascularisation [8, 9]] by measuring perfusion and diffusion respectively. Semi-quantitative and heuristic DCE parameters such as maximal enhancement of contrast (ME), time to peak (TTP), maximal initial slope of enhancement (MIS) and time intensity curves (TIC) have shown their value in determining disease activity in several studies in JIA [10, 11] and rheumatoid arthritis (RA) patients [12–15]. The DWI-derived apparent diffusion coefficient (ADC) is an excellent marker for cellularity in solid tumours [16]. The ADC might also be able to detect microstructural synovial alterations indicative of inflammation as seen in JIA. We hypothesise that, due to increased vascularisation into the synovium, hypertrophied synoviocytes and increased vascular permeability, diffusion of water molecules in the intra- and extracellular compartments of the synovial membrane will increase, resulting in a higher ADC in patients with synovial inflammation. Measuring diffusion in the synovial membrane is novel, with only two studies reporting ADCs of the synovium in patients with JIA [17, 18]. Both studies did not include control patients or patients with inactive JIA. Because of these limitations and the scarcity of DWI data in JIA, it is relevant to further expand DWI research. In addition, the lack of need for gadolinium-based contrast agent (GBCA) administration in DWI is of particular relevance, especially in the light of the recent findings of gadolinium deposition in the brain of patients who repeatedly underwent contrast-enhanced MRI [19–23].

The aim of this study is [1] to assess DCE and DWI parameters in the synovium for their ability to discriminate MRI-active JIA from MRI-inactive JIA and [2] to study the correlation between DCE and DWI parameters of the synovium in JIA. We hypothesise that both DCE parameters and DWI-derived ADC can discriminate active from inactive JIA. Second, we hypothesise that DCE and DWI correlate when assessing the synovium of patients with JIA.

## Materials and Methods

### Patients

Patients participating in this cross-sectional study were admitted for an MRI scan in the Academic Medical Center, Amsterdam, The Netherlands, between February 2013 and December 2014. The institutional review board approved the conduct of this study. Written informed consent was obtained prior to MRI acquisition: in children aged 12 or older both the child and the parents gave their consent; in children younger than 12 years, only parental consent was obtained. Inclusion criteria for participation were [1] age between 8 and 18 years, [2] an established diagnosis of JIA according to the International League of Associations for Rheumatology (ILAR) criteria [24] and [3] current or previous knee complaints (pain, swelling, limitation of motion) in the scope of JIA. Patients were excluded if intra-articular steroid injection or trauma in the knee had occurred in the past 6 months or if general contra-indications for MRI scanning existed (e.g. claustrophobia or a pacemaker). Demographic information, clinical data and laboratory measurements from patients were obtained prior to MRI by a paediatric rheumatologist.

### MRI

All patients underwent knee MRI on a Philips 1.0-T open-bore scanner (Panorama HFO, Philips Medical Systems, Best, The Netherlands). The scanning protocol consisted of a sagittal, coronal and transverse T2-weighted SPIR, sagittal T1-weighted TSE, transverse single-shot echo planar imaging (EPI) T2-weighted DWI and three-dimensional T1-weighted gradient-echo DCE followed by two post-contrast sequences: a transverse T1-weighted SPIR and a sagittal T1-weighted TSE. A bolus of the GBCA Gadovist (0.1 mmol/kg body weight gadobutrol, Bayer Healthcare, Berlin, Germany) was administered 45 s after initiation of the DCE at an injection rate of 3 ml/s followed by a saline chase of 15 ml with an xequal injection rate. After acquisition of all sequences, an ADC_50-600_ map was created by the scanner software using b-values of 50 and 600. B-values <50 were not used for the ADC map to omit diffusion signal introduced by vascular flow. Additional MRI parameters are listed in Table 1.

**Table 1:** MRI scanning protocol. A dedicated knee coil was used. Patients were placed in supine position with their knee centrally located in the coil and the eight listed sequences were acquired chronologically. During the sixth sequence (DCE), gadolinium-based contrast agent was administered

	IV Gd	Repetition time (ms)	Echo time (ms)	Flip angle	Field of view	Voxel size (mm)	Slice thickness (mm)	Recon. matrix	TSE factor	NSA
Sagittal T2 SPIR	-	2800-4500	50	90°	150 × 150 × 92	0.5 × 0.6	4	445	14	3
Coronal T2 SPIR	-	2800-4500	60	90°	150 × 150 × 92	0.5 × 0.6	4	480	13	3
Axial T2 SPIR	-	2800-4500	50	90°	150 × 150 × 104.8	0.5 × 0.55	4	480	15	3
Axial T2 EPI DWI	-	6282-8164	98-114	90°	180 × 160 × 63	1 × 1	3.5	256	-	8
Sagittal T1 TSE	-	450-650	10	90°	150 × 150 × 92	0.45 × 0.63	4	480	6	3
Axial T1GE DCE	+	9-10	6.9	30°	180 × 160 × 63	1 × 1	3.5	256	25 (TFE)	1
Sagittal T1 TSE	+	450-650	10	90°	150 × 150 × 92	0.45 × 0.63	4	480	6	3
Axial T1 SPIR	+	400-750	10	90°	150 × 150 × 104.8	0.55 × 0.77	4	480	6	2

IV Gd = intravenous GBCA injection,, Recon. = reconstruction, NSA = number of signal averages, TSE = turbo spin echo, TFE = turbo field echo, SPIR = spectral presaturation inversion recovery, EPI = echo planar imaging, GE = gradient echo.

### Conventional MRI assessment

**Fig. 1:**
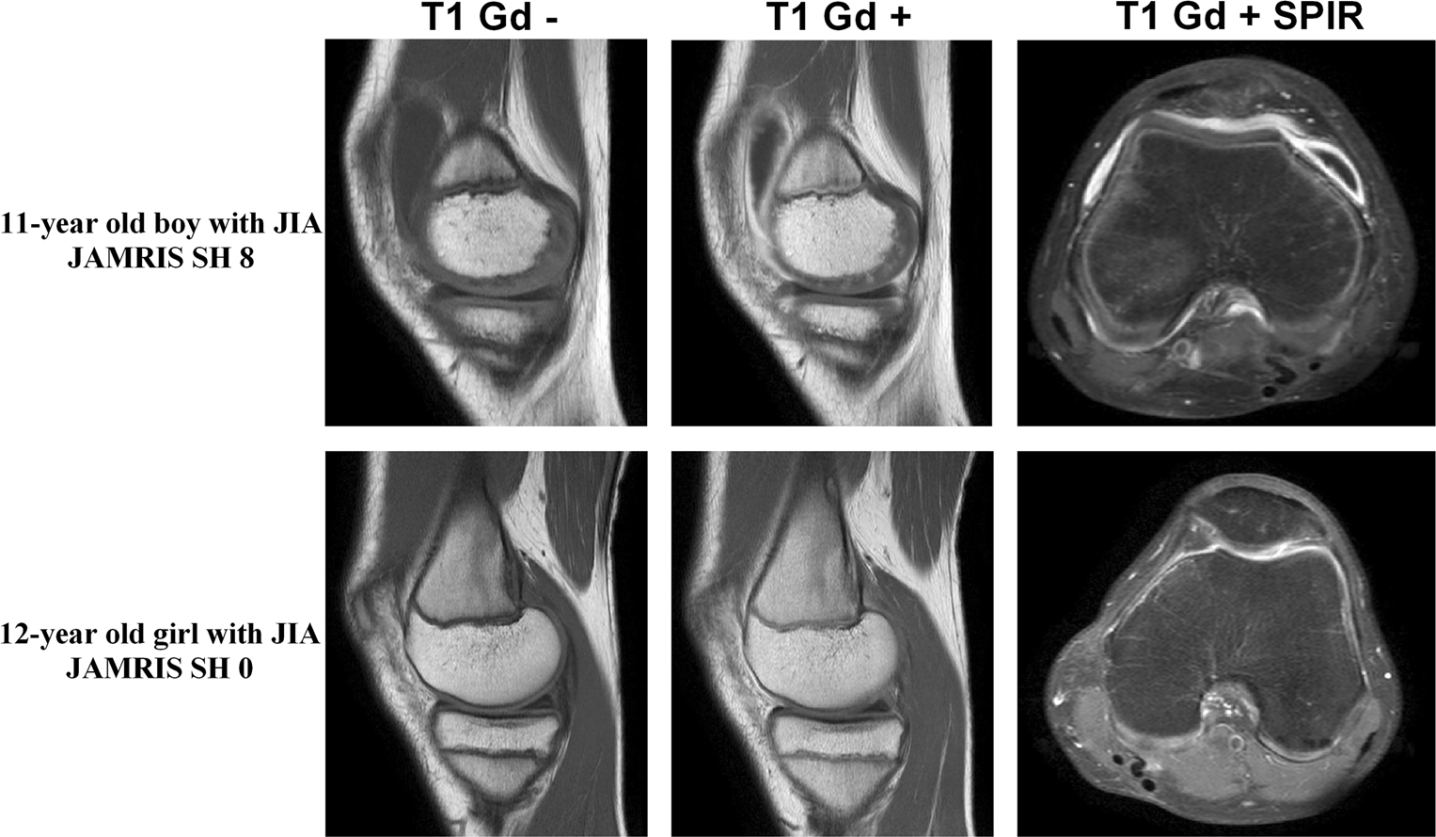
MRI example of two patients using a sagittal T1 pre- and post-contrast image and an axial T1 SPIR post-contrast image. MRI-active disease: the *upper row* represents an 11-year old boy with JIA who had synovial hypertrophy in multiple knee compartments, resulting in a total JAMRIS (Juvenile Arthritis MRI Scoring System) synovial hypertrophy (SH) score of 8. Note the enhancing and thickened synovial membrane in both the sagittal T1 post-contrast slice and the axial T1 SPIR. MRI-inactive disease: the *lower row* represents a 12-year old girl with JIA with no synovial hypertrophy in the knee (JAMRIS SH 0)

Patients were divided in two groups (MRI-inactive and -active JIA) based on the presence or absence of synovial inflammation on MRI as defined by JAMRIS in which measurements of synovial membrane thickness are carried out in six knee compartments, as described before in detail [7]. A total score of 0 for synovial hypertrophy corresponds to no synovial inflammation (MRI-inactive JIA); a score of 1 or higher for synovial hypertrophy is perceived as synovial inflammation (MRI-active JIA). Figure 1 demonstrates representative MR images of a patient with MRI-inactive JIA and a patient with MRI-active JIA. All MRIs were scored by one experienced reader (RH, 7 years experience with MR imaging in the knee) who was blinded for clinical history, current symptoms and clinical examination of the patients.

### DCE and DWI

**Fig. 2:**
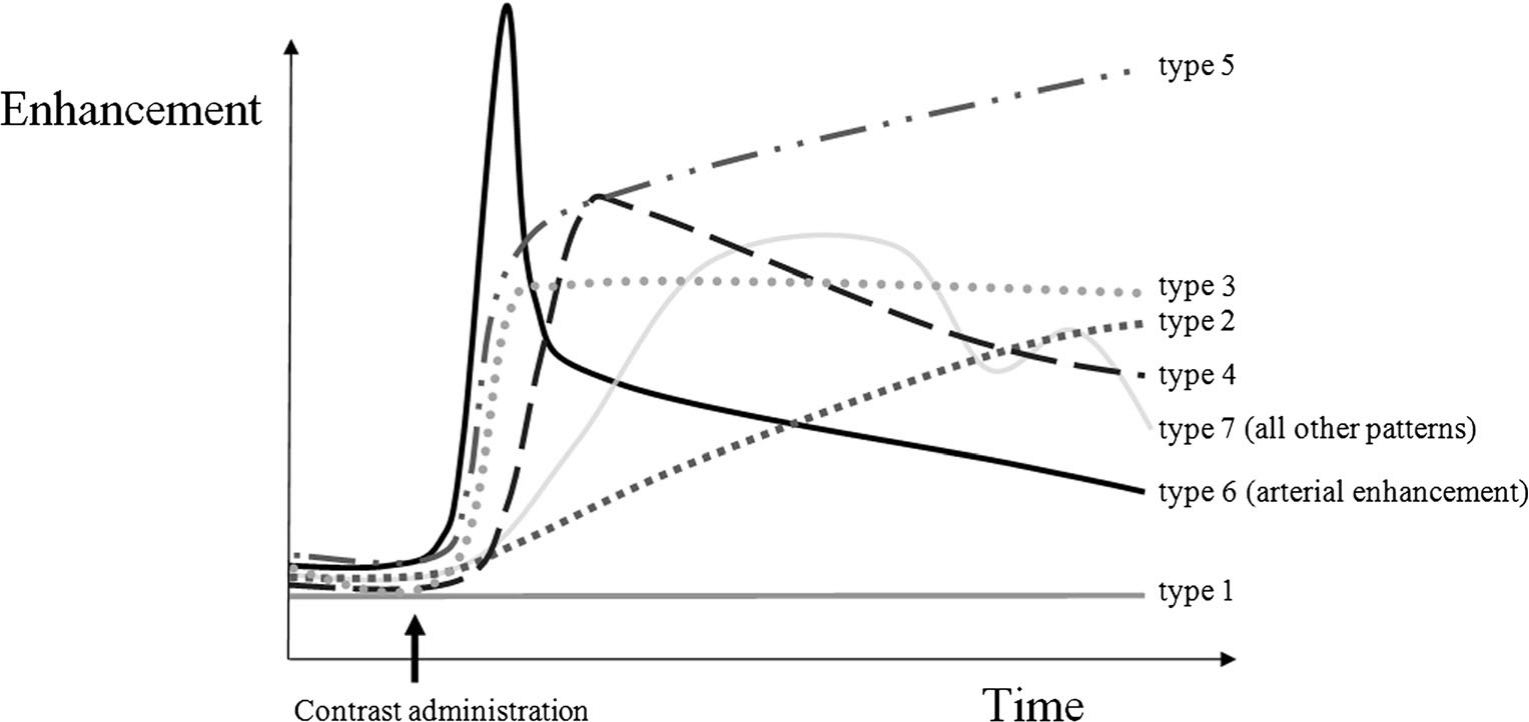
Visualisation of the seven-time intensity curve (TIC) shapes that were used in the TIC shape analysis. A detailed explanation of the TIC shape analysis can be found in references [25, 26]

In-house developed software [Dynamo [25]] developed in MATLAB (version 2012b, The MathWorks, Inc., Natick, MA, USA) was used to manually draw ROIs in the synovial membrane. ROIs were positioned in the same compartments as used for the JAMRIS with the exception of the supra-patellar compartment since this area was not within the field of view of the DCE and DWI scans. Conventional MR images were available as anatomic reference for selection of the synovium. Joint effusion, if present, was not included in the ROI: only synovial tissue was selected. Per location, one ROI was drawn on the last dynamic image (i.e. contrast enhanced) of the DCE. Second, ROIs were redrawn similarly on the ADC_50-600_ map. If no synovium was visible in one of the JAMRIS compartments on the DCE image or ADC map, no ROI was drawn. Drawing of all ROIs was done by one reader (AMB, 4 years experience with knee MR imaging) who was blinded for the JAMRIS score, clinical history, current symptoms and clinical examination of the patient. The drawing procedure was supervised by RH and a musculoskeletal radiologist (MM, 20 years experience in musculoskeletal radiology). To test the reproducibility of the ROI drawing, the same reader repeated the ROI drawing in the first five patients after 1 week. Thereafter, Dynamo extracted quantitative data from the ROIs. For the DCE images, median values of ME, TTP and MIS were calculated. Moreover, TIC shapes (Fig. 2) were classified into different TIC types (1–7) using a pixel-by-pixel approach according to a method as previously described [25, 26]. In short, type 1 represents unenhanced pixels and type 2 slowly enhancing pixels; type 3, 4 and 5 all represent quickly enhancing pixels followed respectively by a plateau, washout or further enhancement; type 6 represents arterial enhancement and type 7 all other patterns. We calculated the total number of pixels classified as each of the seven curve shapes. Next, we calculated the total number of pixels classified as each of the seven curve shapes together with the relative occurrence of each of these TIC shapes (number of pixels per shape/total number of pixels in the ROI). From the ADC map, Dynamo extracted the median ADC of the ROI-captured synovium. Per patient, all ROI-derived measures from the JAMRIS compartments were averaged resulting in mean synovial parameters per individual. Combining the data of all compartments into mean synovial parameters ensures that every patient’s joint is considered as one entity; this fits with the concept that inflammation in a joint can be active in one area but relatively silent in other areas [27, 28].

### Statistical analyses

IBM SPSS Statistics version 22.0 (IBM Corp., Armonk, NY, USA) was used to perform statistical testing. Assumptions of normality were checked by visual inspection of the data in histograms. If the data had a non-normal distribution, non-parametric tests were used and medians and the interquartile range (IQR) were reported. For all tests, a *p*-value ˂ 0.05 was considered statistically significant. Chi-square tests and Mann-Whitney U tests were used to compare patient characteristics between patients with MRI-inactive and -active JIA.

### MRI-inactive disease vs. MRI-active disease

Mann-Whitney U test was used to compare DCE and DWI-derived data (ME, TTP, MIS, TIC and ADC) between patients with MRI-inactive and -active JIA.

### Correlation between DCE and DWI

Spearman’s rank correlation coefficient was used to study the correlation between DCE parameters and the ADC.

To analyse the reliability of ROI drawing, the correlation between the first and second measurements was analysed in the first five patients using a two-way mixed intra-class correlation coefficient (ICC) based on the average measures. ICC and Spearman correlation was regarded as not correlated if the ICC or ‘*r*’ was in between 0.0 and 0.19, weakly correlated if 0.20 -0.39, moderately correlated if 0.40-0.59, strong if 0.60-0.79 and very strong if ICC was between 0.80 and 1.00.

## Results

### Patients

Forty children with JIA were included in this study. Five patients were excluded: four patients from the MRI-inactive JIA group were excluded because the synovium was too thin to draw ROIs; one patient was excluded because of an incomplete DCE sequence resulting from discomfort and subsequent movement during contrast administration. The remaining 35 JIA patients were subdivided in two groups based on the JAMRIS for synovial hypertrophy (SH): 17 patients (49%) had MRI-inactive JIA (JAMRIS SH of 0); 18 patients (51%) had MRI-active JIA (median JAMRIS SH 4, IQR 2-5).

**Fig. 3:**
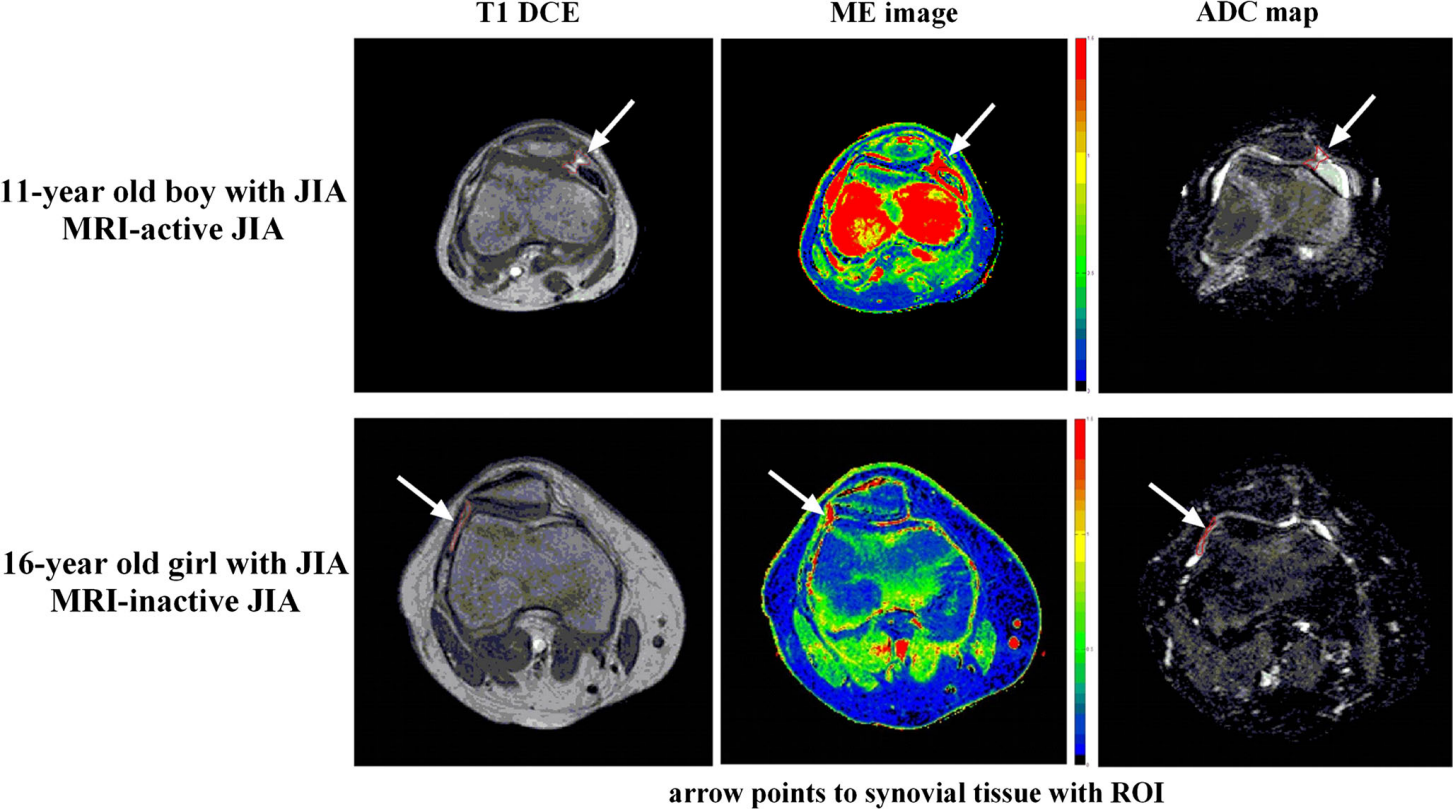
Example of a T1 dynamic contrast-enhanced image and parametric maps (maximum enhancement (ME) and apparent diffusion coefficient (ADC)) of an MRI-active JIA patient (*upper row*) and MRI-inactive JIA patient (*lower row*) with demonstration of region of interest (ROI) placement. Arrows point to the ROIs. ROIs were placed in synovial tissue. Joint effusion (if present) was not included in the ROI, as can be seen in the T1 DCE image of the 11-year old boy with MRI-active JIA

The median age of the children was 13.1 years (IQR 11.4–15.7 years) and 25 of 35 patients (71%) were female. Patient characteristics and disease activity parameters of MRI-inactive and -active JIA patients are shown in Table 2. No statistically significant differences between the two groups were observed regarding those clinical parameters. In active patients, the median number of ROIs per patient was 5 (IQR 5-5); for inactive JIA, the median number of drawn ROIs per patient was 4 (IQR 3-5). In total, 151 ROIs were drawn. An imaging example of ROIs in an MRI-active patient and MRI-inactive patient is shown in Fig. 3.

**Table 2:** Patient characteristics of the MRI-inactive and -active JIA patients. Non-parametric tests were used to study homogeneity of the two groups. *P*-values for all patient characteristics are displayed in the last column. Note that no statistically significant differences were seen between MRI-inactive and -active JIA patients

	Group 1 MRI-inactive JIA (*n* = 17)	Group 2 MRI-active JIA (*n* = 18)	*p*-value
% Females (no. of females)	77% (13)	67% (12)	0.711^a^
Median age in months (IQR)	163 (143-196)	150 (129-177)	0.13^b^
% ILAR subtype (no.)			
Oligo-persistent	53% (9)	56% (10)	0.88^c^
Oligo-extended	6% (1)	6% (1)	1.00^a^
Poly-RF +	12% (2)	0% (0)	0.23^a^
Poly-RF –	24% (4)	28% (5)	1.00^a^
ERA	0% (0)	11% (2)	0.49^a^
Psoriatic	6% (1)	0% (0)	0.49^a^
Median ESR (IQR)	6 (2-8)	5 (2-8)	0.48^b^
Median CRP (IQR)	0.4 (0.0-3.6)	0.3 (0-0.5)	0.29^b^
Median number of active joints (IQR)	0 (0-4.0)	1 (0.75-1.25)	0.55^b^
Median Physician Global Assessment of disease activity (0-100) (IQR)	4 (0-18)	11 (7-21)	0.08^b^

IQR = interquartile range, ILAR = International League Against Rheumatism, RF = rheumatoid factor, ERA = enthesis-related arthritis, ESR = erythrocyte sedimentation rate, CRP = C-reactive protein

^a^Fisher’s exact test

^b^Mann-Whitney U test

^c^Pearson chi-square test

### MRI-inactive disease vs. MRI-active disease

**Fig. 4:**
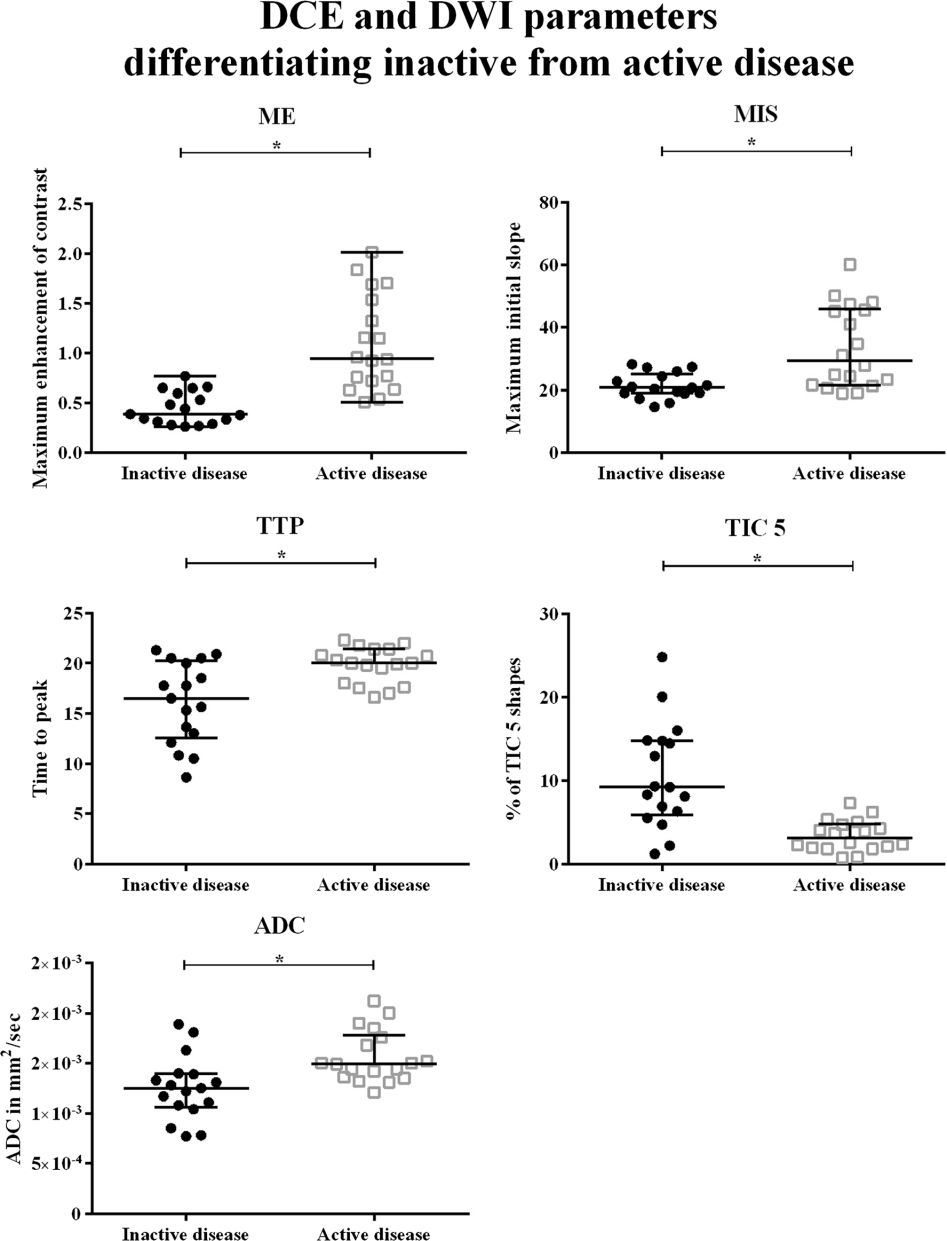
*Boxplots* of dynamic contrast-enhanced (DCE) and diffusion-weighted imaging (DWI) parameters that were significantly different in MRI-inactive JIA when compared to MRI-active JIA. The *y*-*axis* displays a DCE or DWI parameter (ME, MIS, TTP, TIC5 or ADC). The *x*-*axis* is divided in two: first, MRI-inactive patients are displayed (*filled circles*); second patients with MRI-active JIA indicated by a *grey*, *open square*. *= *p*-value < 0.05

ME, MIS, TTP, TIC 5 and ADC were significantly different when MRI-inactive and -active JIAs were compared. Medians and IQR of all parameters are shown in Table 3. Also, the 95% confidence interval of the difference in the medians is given. Higher percentages of TIC 5—quickly enhancing pixels followed by further enhancement—were seen in MRI-inactive JIA (Fig. 4). Lower ME, MIS, TTP and ADC were found in MRI-inactive JIA as compared to MRI-active JIA, as shown in Fig. 4.

**Table 3:** Median values of DCE parameters and ADC in patients with MRI-inactive and -active JIA. Interquartile range is given in between brackets. The 95% confidence interval of the difference between medians (MRI-active versus MRI-inactive JIA) is given in the third column. The associated *p*-value of the difference between the medians as calculated by the Mann-Whitney U test is listed in the last column. *Statistically significant correlation (*p*-value < 0.05)

Variable	Median MRI-inactive JIA (IQR)	Median MRI-active JIA (IQR)	95% CI of difference between medians	*p*-value
Maximum enhancement	0.39 (0.30-0.62)	0.95 (0.70-1.57)	0.32, 0.88	0.000*
Maximum initial slope	20.80 (18.96-25.19)	29.40 (21.57-45.99)	2.56, 21.14	0.002*
Time to peak	16.50 (12.55-20.25)	20.00 (17.90-21.40)	0.94, 6.17	0.005*
% TIC 2 shapes	54 (41-70)	61 (53-72)	-5, 21	0.195
% TIC 3 shapes	3 (1-5)	8 (1-32)	0.2, 26	0.062
% TIC 4 shapes	4 (1-10)	2 (1-5)	-5, 0.5	0.195
% TIC 5 shapes	9 (6-15)	3 (2-5)	-11, -3	0.000*
Apparent diffusion coefficient	1.25 × 10^3^ (1.06-1.39)	1.49 × 10^3^ (1.36-1.78)	0.11, 0.53	0.001*

TIC = time intensity curve, IQR = interquartile range

### Correlation between DCE and DWI

**Fig. 5:**
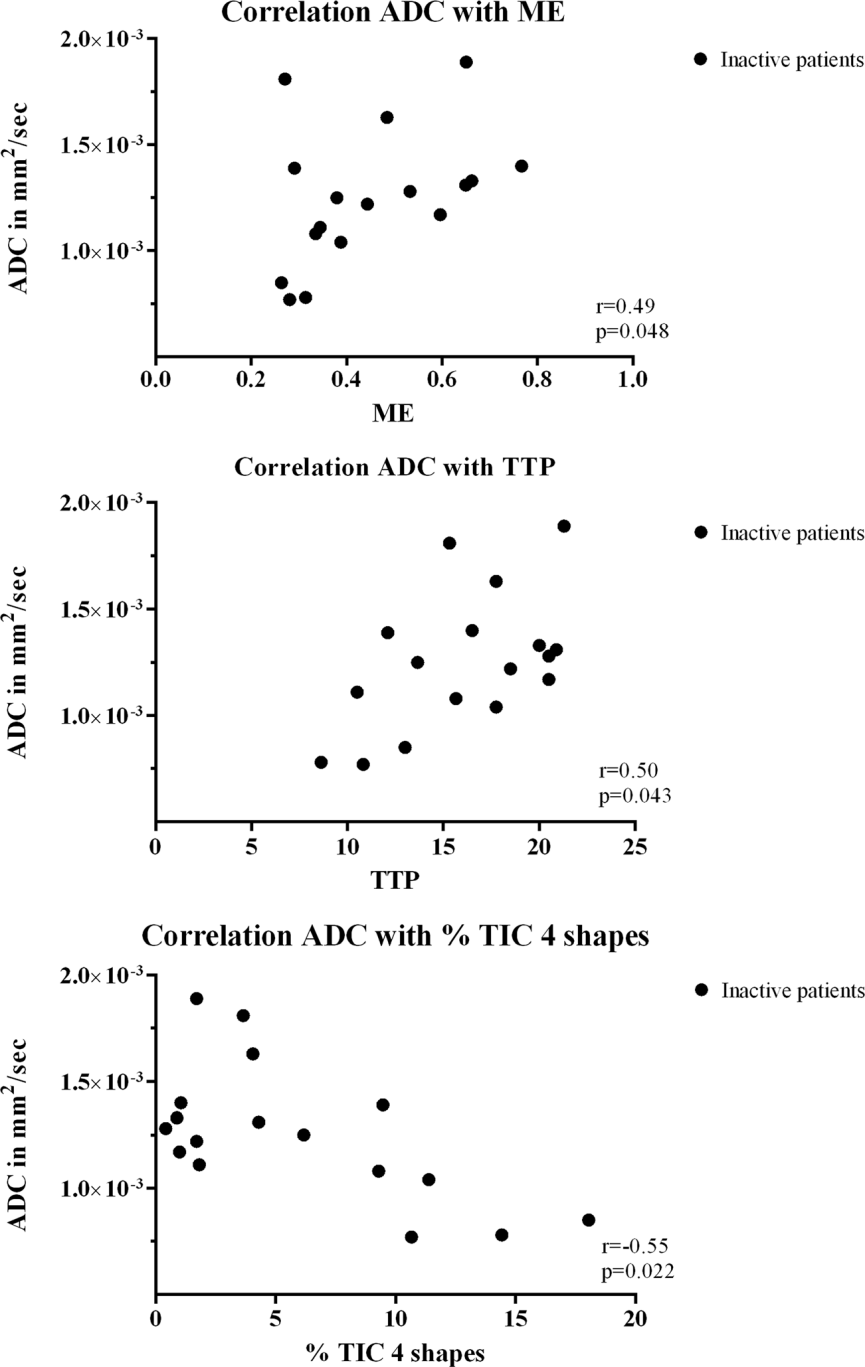
*Scatter plot* displaying the correlation between dynamic contrast-enhanced (DCE) parameters and apparent diffusion coefficient (ADC) in patients with MRI-inactive JIA. The *y*-*axis* represents ADC in mm^2^/s; the *x-axis *displays the DCE parameters (ME, TTP and % of TIC 4 shapes). Correlation coefficient '*r*' and the associated *p*-value are displayed within the right corner of each scatter plot. Only statistically significant correlations are shown

**Fig. 6:**
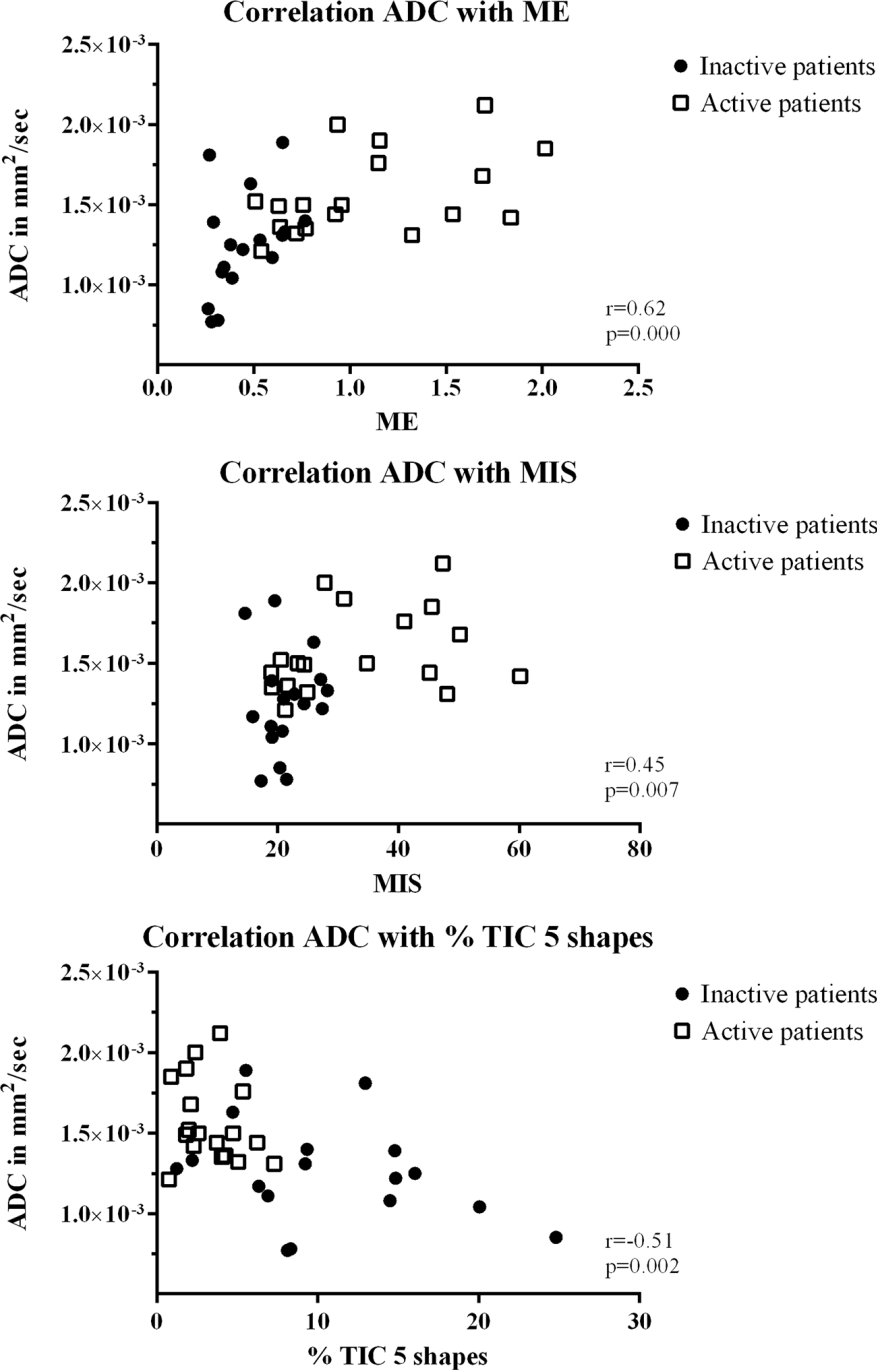
*Scatter plot* displaying correlation between the dynamic contrast-enhanced (DCE) parameters and apparent diffusion coefficient (ADC) in all patients (MRI-inactive and -active JIA). The *y*-*axis* represents ADC in mm^2^/s; the *x*-*axis* displays the DCE parameters (ME, MIS and % of TIC 5 shapes). MRI-inactive patients are represented by *filled circles*, MRI-active patients by a *grey*, *open square*. Correlation coefficient '*r*' and the associated *p*-value are displayed within the right corner of each scatter plot. Only statistically significant correlations are shown

In MRI-inactive JIA, ME positively correlated with ADC with *r* = 0.49 and *p* = 0.048. Also, TTP positively correlated with ADC with *r* = 0.50, *p* = 0.043, and TIC 4 inversely correlated with ADC with *r* = -0.55, *p* = 0.022 (Fig. 5). ME, MIS and TIC 5 significantly correlated to ADC (0.62, 0.45, -0.51 respectively, all *p* < 0.05) when assessing MRI-active and inactive JIA together (Fig. 6). Other correlations between DCE parameters and ADC for inactive and active patients did not reach statistical significance. Correlation coefficients and associated *p*-values of all parameters are given in Table 4.

**Table 4:** Correlation of all DCE parameters with ADC, analysed with all patients together (MRI-inactive and -active JIA) and in MRI-inactive and -active JIA separately. Correlation coefficient *r* is given, as well as the associated *p*-value. *Statistically significant correlation (*p*-value < 0.05)

Variable	‘*r*’ of all patients	*p*-value	‘*r*’ of inactive JIA	*p*-value	‘*r*’ of active JIA	*p*-value
Maximum enhancement	0.62	0.000*	0.49	0.048*	0.39	0.106
Maximum initial slope	0.45	0.007*	0.20	0.451	0.29	0.250
Time to peak	0.33	0.056	0.50	0.043*	-0.33	0.184
% TIC 2 shapes	0.28	0.103	0.46	0.064	0.06	0.826
% TIC 3 shapes	0.20	0.247	-0.21	0.411	0.10	0.705
% TIC 4 shapes	-0.17	0.335	-0.55	0.022*	0.36	0.143
% TIC 5 shapes	-0.51	0.002*	-0.32	0.213	-0.27	0.278

TIC = time intensity curve

### Reliability of ROI drawing

The first and second ROI measurements were compared in the first five patients to assess the reliability of ROI drawing. Concerning DCE parameters, very strong correlations were observed for ME, MIS and TIC 3-5 (ICCs 0.80-0.98, all *p* < 0.05). A moderate non-significant ICC was found for TTP (ICC 0.41, *p* = 0.269) and TIC 2 (ICC 0.58, *p* = 0.127). For DWI-derived ADC, a very strong correlation was observed (ICC 0.86, *p* = 0.014). ICC per parameter, 95% confidence interval (CI) and associated *p*-values are listed in Table 5.

**Table 5:** Intra-class correlation coefficient (ICC) of first and second ROI measurements for all DCE parameters to assess the reliability of the region of interest (ROI) drawing. Within brackets, the 95% confidence interval (CI) of the ICC is given. The last column represents the *p*-value of the found correlation. *Statistically significant correlation (*p*-value < 0.05)

Variable	ICC (95% CI)	*p*-value
Maximum enhancement	0.86 (0.31-0.97)	0.011*
Maximum initial slope	0.95 (0.75-0.99)	0.001*
Time to peak	0.41 (-2.85-0.89)	0.269
% TIC 2 shapes	0.58 (-0.75-0.91)	0.127
% TIC 3 shapes	0.98 (0.89-1.00)	0.000*
% TIC 4 shapes	0.85 (0.17-0.97)	0.016*
% TIC 5 shapes	0.80 (0.11-0.96)	0.024*
Apparent diffusion coefficient	0.86 (0.21-0.97)	0.014*

ICC = intra-class correlation coefficient,, IC = confidence interval, TIC = time intensity curve

## Discussion

Detection of synovial inflammation by the functional, non-invasive MRI sequence DWI as demonstrated in this study shows the potential of DWI as an imaging biomarker in the assessment of disease activity of the joints in rheumatologic diseases. Omitting intravenous cannula placement is patient-friendly, and rare but possible adverse reactions to GBCAs are avoided [29, 30]. In the light of the current discussion on gadolinium depositions in the brain after repeated use of GBCAs during MRI [19–23], it is even more important, especially in children, to focus on non-contrast sequences.

Reports on the use of DWI in arthritis are scarce. This is the first study to present data on DWI in the knee of patients with JIA comparing active and inactive disease. We compared DCE parameters with the ADC, which is novel in rheumatic disease, in both JIA and RA. Semi-quantitative and heuristic DCE analyses were used instead of a pharmacokinetic DCE model since these methods are simple to implement, robust in their performance and have no stringent requirements on the data, which would make implementation in a clinical setting feasible in practice. We tested the hypothesis that DWI, similar to DCE, is feasible to evaluate synovial inflammation in the knee in children with JIA. We found that DWI-derived ADC, next to several DCE parameters, revealed significantly different values in MRI-inactive JIA compared to MRI-active JIA. Moreover, looking at the correlation between DCE and DWI, we found a strong correlation between ME and ADC when assessing MRI-active and -inactive patients together and when looking at MRI-inactive patients. Considering the robustness of ME as an indicator of synovial inflammation in both our study and earlier studies on arthritis [10, 11] as well as the presence of increased ADC values in MRI-active JIA patients (ADC 1.49 × 10^3^ mm^2^/s versus 1.25 × 10^3^ mm^2^/s in MRI-inactive JIA, *p* = 0.001), we conclude that ADC is a promising imaging marker of synovial inflammation. Nevertheless, our analysis failed to show a significant correlation between ME and ADC in MRI-active patients (*n* = 18). This might be due to the relatively low number of patients and consequently the large influence of some outliers in ADC values >1.70 × 10^3^ mm^2^/s, as can be seen in Fig. 6.

Previous to this study, two articles described synovial ADC values of JIA patients with knee arthritis. In a study by Neubauer et al. focusing on DWI in musculoskeletal lesions, 12 children with synovial inflammation were described [17]. The mean ADC value in lesions was 2.12 × 10^3^ mm^2^/s, a much higher value than we found in the MRI-active JIA group (1.49 × 10^3^ mm^2^/s). This difference could be ascribed to different post-processings of the diffusion images. We used blood flow-insensitive ADC maps (ADC_50-600_ maps) [31], including only moderate or high b-values, whereas the Neubauer study included low b-values in creating ADC maps [17]. In another study, ADC of patients with active JIA was also higher (1.92 × 10^3^ mm^2^/s) [18] than the value we found in this study; however this result was based on data derived from only eight synovial ROIs of eight patients. In our study, the median ADC of patients with MRI-active disease is determined by using 90 ROIs from different knee compartments (5 ROIs per patient × 18 patients). Unfortunately, the ADC value of our MRI-inactive JIA group cannot be compared to data of these studies because the authors did not describe JIA patients with inactive disease.

Looking into our results in more detail we assume that DCE and DWI parameters reflect different physiological processes during inflammation of synovium, since not all DCE parameters correlate with the ADC: for time to peak, % of TIC 2 (slowly enhancing pixels) and TIC 3 shapes (quickly enhancing voxels followed by an enhancement plateau), no correlation with ADC was found. This might be due to an essential technical difference between DCE and DWI. In DCE, paramagnetic GBCA disseminates from arteries to the extravascular extracellular space but it does not cross cell membranes [32]. In contrast, in DWI, water molecules diffuse through cell membranes. For instance, in DWI, when we inspect the synovium, we essentially examine diffusion inside and into the synoviocytes along with the diffusion in their surroundings, whereas with DCE we study GBCA uptake in the extracellular space surrounding synoviocytes. An alteration of the ADC may occur when the number of synoviocytes increases or when synoviocytes have become hypertrophic as a result of inflammatory stimuli. Changes in the microenvironment surrounding synoviocytes, e.g. hypervascularisation and the influx of inflammatory cells in JIA and RA[8, 9], are likely to affect both DWI and DCE parameters. In our study we observed significantly higher ADC in patients with MRI-active JIA as compared to MRI-inactive JIA. Hence, we hypothesise that newly formed semipermeable vessels and hypertrophic synoviocytes contribute to this increased diffusion. However, without histological proof, it is difficult to directly correlate functional MRI parameters to changes in the microenvironment of the synovium. Nevertheless, notwithstanding the fact that definite answers are not yet available, in this research setting both techniques distinguished active from inactive JIA, which is the major clinical topic for patients and paediatric rheumatologists in clinical management of JIA.

Several limitations of this study need to be addressed. First of all, there are some drawbacks regarding the ROI method that was used. We selected multiple (3 to 5) synovial ROIs per patient; thus, the entire volume of the synovium was not studied. This could have led to selection of a non-representative sample of the total synovial volume. In addition, it was not possible to extrapolate the ROI from DCE images to the ADC map because of distortions present in the EPI DWI. Therefore, the ROI needed to be redrawn on the ADC map. This potentially could produce small differences in selected voxels in the ROIs of DCE images as compared to selected voxels in the ROIs of the ADC map. By providing the reader access to the conventional MRI sequences, including contrast-enhanced T1, redrawing of the ROI on the ADC map was as accurate as possible. To estimate the possible inaccuracy of this ROI redrawing, the reader also placed ROIs on the first five patients twice and we analysed the intraclass correlation coefficient between the first and second measurement. We believe the ICCs that we report indicate a fairly good correlation and therefore that the hypothetical small variance of selected voxels did not result in a meaningful deviation in functional MRI parameters. Nevertheless, the first and second ROI measurements were performed 1 week from each other. A more prolonged interval might be advisable. A second limitation was the utilisation of a 1.0-T open-bore scanner. The advantage of the open-bore scanner, namely its child-friendliness, might not outweigh the increased temporal resolution and higher signal-to-noise ratio that is obtained using a stronger magnetic field. Therefore, further research on the thin synovial membrane should be carried out using higher magnetic field strengths, preferably on 3.0-T scanners. Another limitation was the absence of ROI placement in the supra-patellar recess due to the field of view, which did not include the supra-patellar recess in DCE and DWI sequences.

In conclusion, both DWI and DCE can visualise active synovial disease in the knee. DWI is potentially powerful in differentiating active from inactive JIA in a non-invasive manner. Exploration on higher field strengths is needed to confirm the feasibility and study accuracy of this patient-friendly contrast-free sequence in children with JIA.
